# Informal creditors and sovereign debt restructuring

**DOI:** 10.1007/s41775-023-00158-z

**Published:** 2023-02-24

**Authors:** Sayantan Ghosal, Dania Thomas

**Affiliations:** University of Glasgow, Glasgow, United Kingdom

**Keywords:** Debt, Restructuring, Default, Welfare, Ex ante, Interim, Elite, Non-elite, Payoffs, Non-contractible, F34, D72

## Abstract

A conventional view of sovereign debt restructuring suggests that costly sovereign debt restructuring is required to lower the interest rate charged on it. In the presence of a negative external shock, under certain conditions, we show that (a) debt restructuring leads to interim social welfare gains and ex ante efficiency gains, (b) participation by citizens will lead to efficient debt restructuring. Using our results, we discuss provide a normative case for the proposed UNCTAD Roadmap.

## Introduction

Even before the global negative shock resulting from the Covid 19 pandemic, a report by the World Bank (2020) noted that total developing country debt in 2018 registered an increase of 54 percentage points of GDP since 2010. The Institute of International Finance estimated that portfolio outflows from emerging market countries amounted to nearly $100 billion over a period of 45 days starting in late February 2020 (IIF 2020). Rapid Financing Initiative was designed to meet the financing needs of several at risk countries in the initial stages of the pandemic as a response to the sudden collapse in capital flows to emerging and developing countries: e.g. the quick agreement to provide financial assistance for 77 Countries under the (see https://www.imf.org/en/Topics/imf-and-covid19/COVID-Lending-Tracker). As the global economy emerges from the pandemic, new problems have emerged, and the Rapid Financing Initiative is no longer be an option. The failure of G-20’s Debt Service Suspension Initiative (only Chad, Zambia, and Ethiopia have applied for relief) is a case in point.

The recent experience of Sri Lanka is a case in point, a combination of bad policy choices and bad luck. Certainly, the pandemic and the conflict between Russia and Ukraine and resulting rise in global commodity prices are all partly to blame and could not have been anticipated. On the other hand, poor policy choices by a corrupt, authoritarian governing elite have also made Sri Lanka uniquely vulnerable. Before 2019, Sri Lanka was self-sufficient in food. The government elected that year banned pesticides so that only organic farming was allowed, resulting in the shutting of tea plantations (a source of export revenue) and shrinking the country’s ability to feed itself (Sri Lanka now imports grains). Together with the damage to tourism from the COVID pandemic and rising global commodity prices, this reduced tax revenues and put more pressure on the Sri Lankan rupee; public finances were further damaged by unsustainable subsidies and unaffordable tax cuts and there were futile attempts (see, for example, https://economynext.com/sri-lanka-spends-us736mn-to-defend-200-to-dollar-peg-as-reserves-for-imports-intensify-90404/) to maintain an unviable currency peg to the US dollar and bad decisions around debt management. Public debt near-tripled (see, for example, https://fortune.com/2022/04/09/sri-lanka-debt-crisis-inflation-rajapaksa-protest-imf-ukraine/) as a percentage of GDP to 104% in three years, while the rupee has jumped from about 200 to the US dollar in early March 2022 to almost 330 by the end of April 2022.

Sri Lanka’s governing elites are not alone in making these kinds of poor policy choices. For example, in the run up to the crisis (see, for example, https://inews.co.uk/news/world/lebanons-swift-devastating-financial-crisis-leaves-residents-feeling-no-future-1448753) that engulfed Lebanon in late 2019 (see, for example, https://www.reuters.com/markets/rates-bonds/lebanons-financial-crisis-how-it-happened-2022-01-23/), the central bank swapped debt held in Lebanese pounds into debt in euros and US dollars while maintaining an unviable currency peg. The fees from these activities generated huge profits for major banks but made the country vulnerable to negative external shocks, such as nervous foreign investors dumping government bonds. This helped to drive down the value of the currency and made debts priced in foreign currency harder to pay back. Just like Sri Lanka, there were protesters on the streets, governing elites waxing eloquently about the need to maintain national unity, and a middle class facing the prospect of being wiped out while millions were pushed into poverty.

The financial crisis in the eurozone in the 2010’s was the result of a similar mix of bad policy choices and bad luck, as was the 1990’s Asian financial crisis (Corsetti et al., 1998a; b) before it, and the Latin American crisis (Devlin & French-Davis, 1995) in the 1980’s.

One common thread that cuts across these different episodes is that a sovereign debt crisis is, typically, followed by an international bailout by the IMF and other bodies, in which the money is conditional on reining in the state through severe cuts to public spending, privatisations and so on. After a gap of a few years, provided the debtor state meets these conditions, borrowing in international capital markets is permitted to resume and the whole cycle repeats itself.

Is there a trade-off between lowering the costs of a sovereign debt restructuring (leading to interim or ex post welfare gains) and the interest rate charged on sovereign debt (linked to ex ante welfare gains)? A conventional view is that (a) default is less likely the higher is the anticipated probability of costly debt restructuring, (b) consequently, the interest rates at which sovereigns can borrow become lower leading to ex ante welfare gains.

The Brookings Report (2013) on sovereign debt restructuring states: ‘If the main problem in sovereign debt is not repudiating debtors and overly tight borrowing constraints, but rather over-borrowing at the front end and procrastination at the back end, then the old trade-off between ex-ante and ex-post efficiency no longer holds, at least within some range. Lowering the costs of debt crises ex post might benefit efficiency ex ante.’ In a recent report, UNCTAD note that “restructurings are often insufficient … This lack of a ‘fresh start’ is often the cause of repeated restructuring episodes and may lead to additional costs to all parties.” In the Brookings report, the complementarity between ex ante and ex post (or interim) efficiency requires the use of a formal sovereign debt bankruptcy procedure. Ghosal and Miller (2003) who make similar point in the presence of creditor coordination failure and sovereign moral hazard.

In contrast, this paper focuses on a different approach, one which puts the needs of citizens first and ensures that political elites are not rewarded for poor policy choices. Bolton and Skeel (2007) highlight the problem of inter-creditor inequity between “formal creditors”, such as a western European pension fund buying sovereign bonds, and “informal creditors” within the debtor state itself, such as pensioners who have contributed to the state social security fund, or workers who have paid into the public insurance system whose claims are based on the social contract between the debtor state and its citizens. Yet in a bailout situation, these informal creditors withstand the worst of austerity, including cuts to social security programmes. Meanwhile, the formal creditors get their money back—albeit with a “haircut” where they lose a proportion of what is owed to them (though such risk is usually already priced into the interest rate at which their money is lent in the first place).

With a focus on ex post efficiency, Guzman and Stiglitz (2016) argue that a debt restructuring process should ‘ensure ‘overall economic efficiency a critical feature of which is ex post efficiency…it should provide the conditions for a rapid and sustained economic recovery’. When sovereign debtors delay debt restructurings and rely on ‘bailouts’ rather than restructurings to avoid bailing-in private creditors, this creates ‘large inter-creditor inequities as only the creditors that get paid with the resources of the “bailouts” benefit while the expected value of the claims of the other claimants (such as the creditors whose debts mature in a longer term, or the workers and pensioners whose wages depend on the capacity of production of the economy that decreases precisely as the consequence of the austerity often associated with those plans) decreases’. The latter category of domestic agents namely workers and pensioners whose wages depend on the capacity of production of the economy that decreases are the “informal creditors” in their paper.

In this paper, we examine the conditions under which, conditional on an economy-wide negative shock, lowering the costs of sovereign debt restructuring also lowers the interest rate charged ex ante on sovereign debt.

In our model, domestic agents are split into two groups, a minority elite and a majority non-elite. The elite can borrow and save in international capital markets (only when there is no default) but their gains from doing so are non-contractible. The non-elite cannot directly participate in international capital markets and their payoffs are claims, part of a social contract, on domestic income generated by investment financed by borrowing from those markets: thus, they “informal creditors” in the market for sovereign debt. In the presence of a negative external shock, under certain conditions, we show that debt restructuring leads to both interim and ex ante welfare gains. However, given the non-contractibility of domestic elite payoffs (except when they voluntarily surrender that right) and the option to save in international capital markets implies that they do not have an incentive to do so. In contrast, the payoffs to domestic non-elites are aligned to the direction of interim and ex ante social welfare gains so in a referendum they would agree, via majority voting, to restructure domestic debt.

What factors determine who, the elite or non-elite, has the decision-making power to restructure debt? When the elite are organised (i.e. can act collectively in their own interests) but the non-elite are not, they will be the decision-makers. For the elite to secede decision-making power to the non-elite to restructure debt, the non-elite must be able to organise itself collectively. Specifically, we show that when the probability of successfully usurping decision-making power, conditional on being fully organised along party lines, is extremely low, no non-elite individual will decide to engage in collective in first place and the elite will retain decision-making power. Moreover, if the individual cost to engage in collective action is high enough, it is either a dominant action for each non-elite individual to not to participate in collective political activity or, in the presence of multiple equilibrium outcomes, the non-elite coordinate on the outcome where no collective activity takes place. In all these cases, the elite retain the decision-making power to restructure debt even if it is interim, and ex ante, efficient to do so.

Our contribution is to show that the participation of informal creditors in the decision to restructure debt to results in both ex ante and interim efficiency gains.

Our analysis relates to recent discussions on “too little, too late” in the context of sovereign debt restructuring. Specifically, our results suggest that when informal creditors directly affected by the decision to restructure debt have a role to play in expediting the decision to restructure debt such workouts are more likely to be conducted soon after negative determinations of debt sustainability and are deep enough to bring the sovereign back to debt sustainability. This point is distinct but complementary to the one made in the Brooking Report referred to above.

The UN Conference on Trade and Development (UNCTAD, 2015) published a debt workout guide that offers a road map for such a process. It proposes referendums at key points in the lead-up to a bailout to ensure that the public see the options and get a chance to vote on them. Our analysis provides a normative for operationalizing such a road map.

Sections 2 and 3 develop an elementary model which enables us to make our point in the simplest possible setting. In Sect. 4, we discuss the policy implications of our analysis. The last section concludes.

## Model

There are three time periods, $t=\mathrm{0,1},2$; a single perishable consumption good in each period.

There are three types of agents: domestic non-elites (informal creditors), mass $1-\alpha , 0<\alpha <\frac{1}{2}$; domestic elites, mass $\alpha$; foreign creditors. Domestic elites and non-elites only care about consumption in the last two periods and have identical linear instantaneous utility $v\left(x\right)=x.$ There is a common discount factor, $0<\delta <1$.

At $t=0$, no domestic agent, either an elite or a non-elite, has endowments of the good in question; domestic output in periods 1 and 2 requires an initial public investment of ${I}^{0}$ which is financed by borrowing from $c$ at a interest rate $r$ to be determined below; without this investment there is no output in either period 1 or 2.

At $t=1$, domestic output is ${Y}^{1}\in \{{Y}_{L}^{1},{Y}_{H}^{1}\}$ with probability $\left\{1-q,q\right\}, 0\le q\le 1$. A fraction $\beta ,0<\beta <1$, appropriated by the domestic elite for their private benefit and the remaining quantity $\left(1-\beta \right){Y}^{1},{Y}^{1}\in \left\{{Y}_{L}^{1},{Y}_{H}^{1}\right\}$, available for further investment, interest repayments on debt and consumption by the domestic non-elites.

At $t=2$, output in period 2 has a stochastic structure that depends on whether or not a further investment is made at $t=1$. Specifically, if an additional public investment of ${I}^{1}$ is made at $t=1$, then output in period 2 is ${Y}^{2}={Y}_{H}^{2}$; otherwise, ${Y}^{2}\in \{{Y}_{L}^{2},{Y}_{H}^{2}\}$ with probability $\left\{p,1-p\right\}, 0<p<1$. As before, a fraction $\beta ,0<\beta <1$, is appropriated by the domestic elite and the remaining quantity available for interest repayments on debt and consumption by domestic non-elite.

At $t=1$, the fraction of output appropriated by the elite can be used either for consumption in that period or for purchasing an asset (requiring a lumpy upfront payment of $\beta {Y}_{L}^{1}$) that which has a private payoff (appropriated entirely by the elite) of ${\beta (Y}_{H}^{2}-{Y}_{L}^{2})$ only when ${Y}^{2}={Y}_{L}^{2}$ or zero otherwise. By purchasing this asset, the elites can guarantee a private payoff of ${\beta Y}_{H}^{2}$ in period 2. However, the option to invest in the asset (labelled “private asset” for later reference) requires access to international capital markets and is available only if there is no default in period 1 and not otherwise.

In any period in which default takes place, there is a direct utility cost of ε′ to the non-elite and their consumption is zero in that period, (b) there is a direct utility cost of ε ≤ ε′ to the elite; however, in contrast to non-elite, the share of domestic output appropriated by the elite is not affected. As this is a finite time-period model, the direct utility costs associated with default are required to ensure that neither the domestic elite nor non-elite have an incentive to strategically default when there are enough domestic resources to service payment obligations linked to sovereign debt.

We make two assumptions throughout the analysis below. These are:

1. $$\frac{{2I}^{0}}{1+q}<\left(1-\beta \right){Y}_{L}^{1}<{I}^{1}+ \frac{{2I}^{0}}{1+q}<\mathrm{min}\{{Y}_{L}^{1},\left(1-\beta \right){Y}_{H}^{1}\}$$;
2. $$\left(1-\beta \right){Y}_{L}^{2}<\frac{3+q}{1+q}{I}^{0}<\left(1-\beta \right){Y}_{H}^{2}$$.

Assumptions (A1) and (A2) ensure that (a) conditional on a negative shock to domestic income in period 1, if interest payments are made, then no further public investment can be made by non-elites on their own resources to ensure a high domestic output in the following period, (b) there will be default at $t=2$ if the domestic output is low.

We are now in a position to state and prove the following proposition which characterises the efficiency implications of two different scenarios of debt restructuring, one where the domestic elite make the decision to restructure debt and the other in which domestic non-elites do so:

### Proposition 1

*Assume *${r}_{f}=1$* and *$\delta >0$. *Suppose assumptions* *(A1)* *and* *(A2)* *hold. Then, there exists *$\overline{Y}>0$*, *$\varepsilon >0$* and* $\overline{\alpha }>0$ *such that whenever *${Y}_{H}^{2}>\overline{Y}$*, *${0<Y}_{L}^{1}-{I}^{1}+ \frac{2+p(1-q)}{2-p(1-q)}{I}^{0}<\varepsilon$*, *$0<\alpha <\overline{\alpha }$* and *$p>\widehat{p}=\frac{2}{3+q}$* it follows that r′* < *r″ and there are both ex ante and interim welfare gains when, conditional on *${Y}^{1}={Y}_{L}^{1}$*, debt is restructure debt in period 1 relative to the situation when it isn’t.*

### Proof

To begin with assume that the following inequalities characterise equilibrium:

1 $$\left( {1 - \beta } \right)Y_{L}^{1} - rI^{0} < I^{1} < Y_{L}^{1} \quad while \quad rI^{0} < \left( {1 - \beta } \right)Y_{L}^{1} \quad and\quad \left( {1 - \beta } \right)Y_{H}^{1} - rI^{0} \ge I^{1}$$

2 $$\left( {1 + r} \right)I^{0} > \left( {1 - \beta } \right)Y_{L}^{2} \quad while \quad \left( {1 + r} \right)I^{0} < \left( {1 - \beta } \right)Y_{H}^{2}$$

(1) and (2) involve restrictions on an endogenous variable $r$: we will verify that these inequalities can be obtained under relevant assumptions on fundamentals in the proof. For the moment, we take these as given. We consider two possible decision-making regimes in relation to restructuring sovereign debt:

Regime A (domestic elites make decisions about whether or not to restructure debt and make public and private investment decisions): in period 2, when ${Y}^{2}={Y}_{H}^{2}$ there is no strategic default (given that $\epsilon >0$) and there is default ${{Y}^{2}=Y}_{L}^{2}$ (given that the elite consumption is not affected by default even though the elite pay a small utility cost $\epsilon$ provided $\epsilon <\beta \left({Y}_{L}^{2}\right)$. In period 1, if ${Y}^{1}={Y}_{H}^{1}$ (as $\epsilon >0$), there is no strategic default. If ${Y}^{1}={Y}_{L}^{1}$, if the elite choose not to default, in the above scenario, the investment of ${I}^{1}$ will not take place so that in period 2, ${Y}^{2}\in \{{Y}_{L}^{2},{Y}_{H}^{2}\}$ with probability $\left\{p,1-p\right\}.$ If the elite chose not to default and use their appropriated share to invest in their private asset their payoff will be $\delta \left[\beta {Y}_{H}^{2}-p\epsilon \right]$ while if the elite choose not to default and do not invest in their private asset, their payoffs will be $\beta {Y}_{L}^{1}+\delta \left[p\beta {Y}_{L}^{2}+\left(1-p\right)\left(\beta {Y}_{H}^{2}\right)-p\epsilon \right]$. So, the elite will choose to not to restructure debt and invest in the insurance if and only if

3 $$\delta p\left[\beta {Y}_{H}^{2}-\beta {Y}_{L}^{2}\right]>\beta {Y}_{L}^{1}\leftrightarrow \delta p\left[{Y}_{H}^{2}-{Y}_{L}^{2}\right]>{Y}_{L}^{1}\leftrightarrow \frac{\delta p\left[{Y}_{H}^{2}-{Y}_{L}^{2}\right]}{{Y}_{L}^{1}}>1$$

If the elite choose to restructure debt in period 1, then it in their interest to make the public investment of ${I}^{1}$ and elite payoffs will be

$$\beta {Y}_{L}^{1}-\left[{I}^{1}-\left(\left(1-\beta \right){Y}_{L}^{1}-r{I}^{0}\right)\right]-\epsilon +\delta \beta {Y}_{H}^{2}={Y}_{L}^{1}-{I}^{1}-r{I}^{0}-\epsilon +\delta \beta {Y}_{H}^{2}.$$

So, by computation, the elite will not choose to restructure debt and invest in the insurance option as long as.

4 $$\varepsilon \left( {1 - p} \right) - Y_{L}^{1} - I^{1} - rI^{0} > 0$$

Regime B (domestic non-elites make decisions about whether or not to restructure debt and make public investment decisions): as before, in period 2, when $${Y}^{2}={Y}_{H}^{2}$$ (as ϵ′ $$>0$$) there is no strategic default and there is default $${{Y}^{2}=Y}_{L}^{2}$$ (the non-elites, using their own resources, do not have enough resources to make debt payments); in period 1, if $${Y}^{1}={Y}_{H}^{1}$$, there is no strategic default (as ϵ′ $$>0)$$. If $${Y}^{1}={Y}_{L}^{1}$$, if the non-elite choose not to restructure debt, the public investment of $${I}^{1}$$ will not take place so that in period 2, $${Y}^{2}\in \left\{{Y}_{L}^{2},{Y}_{H}^{2}\right\}$$ with probability $$\left\{p,1-p\right\}.$$ In this case, their payoff will be.

$$\left(1-\beta \right){Y}_{L}^{1}-r{I}^{0}+\delta \left[-p\epsilon \mathrm{^{\prime}}+\left(1-p\right)\left(\left(1-\beta \right){Y}_{H}^{2}-\left(1+r\right){I}^{0}\right)\right]$$

If the non-elite choose to restructure debt and make the public investment of ${I}^{1}$, their payoff will be.

$$-\epsilon \mathrm{^{\prime}}+\delta \left[\left(\left(1-\beta \right){Y}_{H}^{2}-\left(1+r\right){I}^{0}\right)\right]$$

Therefore, the non-elite will choose to restructure debt in period 1 and make the public investment ${I}^{1}$ iff

5 $$\delta p\left( {\left( {1 - \beta } \right)Y_{H}^{2} - \left( {1 + r} \right)I^{0} } \right) > \left( {1 - \delta p} \right)\varepsilon^{\prime} + \left( {\left( {1 - \beta } \right)Y_{L}^{1} - rI^{0} } \right)$$

Note that, conditional on ${Y}^{1}={Y}_{L}^{1}$, from the perspective of social welfare (using weights $1-\alpha$ and $\alpha$ to aggregate non-elite and elite utility respectively, it is interim efficient to default in period 1 iff.

6 $$\left( {1 - \alpha } \right)\left[ {\delta p\left( {\left( {1 - \beta } \right)Y_{H}^{2} - \left( {1 + r} \right)I^{0} } \right) - \left( {1 - \delta p} \right)\varepsilon^{\prime} - \left( {\left( {1 - \beta } \right)Y_{L}^{1} - rI^{0} } \right)} \right] + \alpha \left[ {\varepsilon^{\prime}\left( {1 - p} \right) - \left( {Y_{L}^{1} - I^{1} - rI^{0} } \right)} \right] > 0$$

Of course, from the conditional on ${Y}^{1}={Y}_{H}^{1}$, it is always interim efficient not to default and make the required investment in period 1.

Next, we check the conditions under which having such referendum would also allow for ex ante welfare gains. So far, in the model, the interest rate on sovereign debt has been taken as given. The interest rate on sovereign debt can be endogenized by requiring that the expected payoff to the (risk neutral) foreign creditor from investing in the sovereign debt be equal to the payoff it would obtain by investing in a risk-free debt instrument. Let ${r}_{f}=1$ denote the risk-free interest rate.

Case 1: Conditional on ${Y}^{1}={Y}_{L}^{1}$, there is debt restructuring. Then, the expected payoff to foreign creditor from investing in the sovereign debt instrument is $(1-q)\left(1+r\right){I}^{0}+q[r{I}^{0}+\left(1+r\right){I}^{0}]$ while payoff from investing in the risk-free asset is ${I}^{0}+2{I}^{0}$ so that, by computation, the interest rate charged is

7 $${r}^{^{\prime}}=\frac{2}{1+q}$$

Case 2: Conditional on ${Y}^{1}={Y}_{L}^{1}$, there is no debt restructuring. Then, the expected payoff to foreign creditor from investing in the sovereign debt instrument is $r{I}^{0}+\left(1-q\right)(1-p)\left(1+r\right){I}^{0}+q\left(1+r\right){I}^{0}$ while the payoff from investing in the risk-free asset is ${I}^{0}+2{I}^{0}$ so that, by computation, the interest rate charged is

8 $${r\mathrm{^{\prime}}}^{\mathrm{^{\prime}}}=\frac{2+p(1-q)}{2-p(1-q)}$$

Finally, we show, by computation, that under the parameter restrictions described in the statement of the result, Eqs. (1–8) are all simultaneously satisfied. First, note that using the expressions for *r*′ and *r*″ in (7) and (8), it immediately follows that (a) assumptions (A1) and (A2) imply the inequalities (1) and (2) hold, and (b) as long as $p>\widehat{p}=\frac{2}{3+q}$, then *r*′ < *r*″. Second, as $\delta >0$, there exists ${\overline{Y}}_{1}>0$ such that whenever ${ Y}_{H}^{2}>{\overline{Y}}_{1}$, it follows that $\delta p\left[{Y}_{H}^{2}-{Y}_{L}^{2}\right]>{Y}_{L}^{1}$ as long as $p>\widehat{p}=\frac{2}{3+q}>0$ so that (3) holds. Third, as $\epsilon >0$ and $\smallint \,\left( {1 - p} \right) = \frac{{\smallint \,\left( {1 + q} \right)}}{3 + q} > \,0$, by continuity, it follows that as long as $0 < Y_{L}^{1} - \,I^{1} + \frac{{2 + p\left( {1 - q} \right)}}{{2 - p\left( {1 - q} \right)}}\,I^{0} < \varepsilon ,\,\varepsilon \left( {1 - p} \right) - \left( {Y_{L}^{1} - I^{1} \, - r^{\prime\prime}I^{0} } \right) > 0$ so that (4) holds. Fourth, as $\delta >0$, there exists ${\overline{Y}}_{2}>0$ such that whenever ${Y}_{H}^{2}>{\overline{Y}}_{2}$, it follows that $\delta p\left( {\left( {1 - \beta } \right)Y_{H}^{2} - \left( {1 + r} \right)I^{0} } \right) > \left( {1 - \delta p} \right)\varepsilon^{\prime} + u\left( {\left( {1 - \beta } \right)Y_{L}^{1} - rI^{0} } \right)$ as long as $p > \hat{p} = \frac{2}{3 + q} > 0$ so that (5) holds. Let $\overline{Y}=\mathrm{max}\{{\overline{Y}}_{1},{\overline{Y}}_{2}\}$. Fifth, as long as (5) holds, when $\alpha =0$, (6) holds so that there exists $\overline{\alpha }>0$ such that when $0<\alpha <\overline{\alpha }$, (6) holds i.e. when debt is restructured conditional on a negative income shock in period 1, there are interim social welfare gains from doing so. Note that the ex-ante and interim payoffs of both the domestic elite and the non-elite are decreasing in the interest rate on sovereign debt. Moreover, the expected payoff to the foreign creditor does not depend on the prevailing interest rate (as it is determined by the no arbitrage constraint). As $p>\widehat{p}=\frac{2}{3+q}$*,*$r\mathrm{^{\prime}} < r^{\prime\prime}$, when debt is restructured conditional on a negative income shock in period 1, there are ex ante efficiency gains from doing so as well. $\square$

The above proposition clarifies that under parameter restrictions which ensure that the negative shock to domestic income is sufficiently severe in both periods 1 and 2 and the expected gains from public investment in period 1 are sufficiently high, there are interim and ex ante welfare gains from debt restructuring in period 1.

However, conditional on a negative shock in period 1, (a) given the non-contractibility of domestic elite payoffs (except when they voluntarily surrender that right) and the option they have to investing in their private asset (which requires access to international capital markets), left to themselves, they do not have an incentive to restructure debt while (b) the payoffs to domestic non-elites are aligned to the direction of interim and ex ante social welfare gains so that in a setting where they decide whether or not to restructure debt, they will do so.

### Political economy of debt restructuring

In this section, we will endogenize that which domestic actors have the power to make the decision to restructure sovereign debt.

What factors determine who, the elite or non-elite, has the decision-making power to restructure debt? When the elite are organised (i.e. can act collectively in their own interests) but the non-elite are not, they will be the decision-makers.

For the elite to secede decision-making power to the non-elite to restructure debt, the non-elite must be able to organise itself collectively.

Following Olson (1965), we assume that successful collective political activity is organised by an organisation (such as a political party or a labour union) whose members are rewarded selectively. Everyone in the non-elite has the choice of becoming a party member; joining the party is costly and becomes a dominant strategy for an individual if and only if the number of other individuals joining the party is greater than a critical threshold. This suggest that there will be two outcomes in equilibrium, one where the non-elite are organised along party or union lines and are able to act collectively to grab the decision-making power over debt restructuring and one in which they remain disorganised and the decision-making power over debt restructuring agents remains vested in the elite. Which outcome prevails will depend on how non-elite individuals solve the underlying coordination problem. A formal analysis is as follows.

To keep matter simple and focus on the key issue, for the remainder of this section, the analysis will be conducted for the situation where $Y^{1} = Y_{L}^{1}$ in a setting where (1–8) hold. Under the assumption that (5) holds, let $\Delta = \delta p\left( {\left( {1 - \beta } \right)Y_{H}^{2} - \left( {1 + r} \right)I^{0} } \right) - \left[ {\left( {1 - \delta p} \right)\varepsilon^{\prime} + \left( {\left( {1 - \beta } \right)Y_{L}^{1} - rI^{0} } \right)} \right] > 0$ i.e. there is a net payoff gain to the non-elite (as a group) when debt is restructured relative to the situation when it is not.

We will make some further assumptions. Let $\pi$ denote the fraction of the non-elite who join the party. It will be assumed that the probability with which the non-elite get to decide whether or not debt is restructured is given by a function $f(\pi$), strictly increasing in $\pi$, with $f\left(0\right)=0$ and $f(1)={f}_{max}\le 1$. Given $\pi \in [0, 1]$, the net payoff gain to a non-elite party member is $f\left(\pi \right)\Delta -c$ where $c$ is the cost of joining the party while the net payoff gain to a non-elite individual who is not a party member is $\gamma f(\pi )\Delta$ where $\gamma \ge 0$ is a small non-negative number close to zero, strictly less than 1.

Clearly if $c\ge f(\pi )\Delta$, then it is a dominant action (strictly dominant when the inequality is strict) for no non-elite individual to join the party and engage in collective action.

Suppose $0\le \gamma f(\pi )<c<f(\pi )\Delta$. Then, there exists a function $\widetilde{\pi }\left(c\right), 0<\widetilde{\pi }\left(c\right)<1$ such that that it is a dominant action for each non-elite individual to join the party if and only if $\pi >\widetilde{\pi }(c)$, where $\widetilde{\pi }(c)$ is the unique solution to the equation $f\left(\pi \right)\Delta -c=\gamma f(\pi )$. As $f(\pi )$ is strictly increasing in $\pi$, $\widetilde{\pi }(c)$ is strictly decreasing as a function of $c$.

When ${\mathrm{c}>\mathrm{f}}_{\mathrm{max}}\Delta$, it is a dominant action for each non-elite individual not to join the party. Hence, only the elite have the decision-making power over debt restructuring.

When ${\mathrm{f}}_{\mathrm{max}}\Delta >\mathrm{c}$, it is a dominant action for each non-elite individual to join the party. Hence, the non-elite have the decision-making power over debt restructuring with probability ${\mathrm{f}}_{\mathrm{max}}$.

When $0\le\upgamma {\mathrm{f}}_{\mathrm{max}}\Delta <\mathrm{c}<{\mathrm{f}}_{\mathrm{max}}\Delta$, (a) if $\pi =0$, it is best response for each non-elite not to join the party, and (b) if $\pi =1$ it is a best response for each non-elite individual not to join the party. Hence, there are two equilibria, one where no non-elite individual joins the party and only the elite have the decision-making power over debt restructuring, and one where all non-elite individuals join the party and the non-elite have the decision-making power over debt restructuring with probability ${\mathrm{f}}_{\mathrm{max}}$.

We will use the notion of a stochastically stable equilibrium (Young (1998)) to select between the two equilibria in the coordination game played by the non-elite. Let $G$ be an arbitrary finite normal form game with a set of $N$ players, an action set ${A}^{i}$ for each player $i=1,..,N$ and a payoff ${u}^{i}:\prod_{i=1}^{N}{A}^{i}\to \mathfrak{R}$. Suppose each player believes that whenever any other player chooses to play a specific action, with probability $\theta , 0<\theta <1$, she ends up choosing some other action in ${A}^{i}$. Let $G(\theta )$ denote the perturbed game. A state in $G(\theta )$ is a profile of actions. For each state, let each player pick a best response to that state in $G(\theta )$ i.e. considering the possibility that other individuals will make a mistake with probability $\theta$. This defines a function $\sigma$ from the set of states to itself. If there are many best responses, then there will be many such functions $\sigma$. When $\theta$ is small enough, let the set of σ′$s$ that remain best responses for all smaller $\theta$ be denoted by$S(G)$. Any$\sigma \in S(G)$. Together with $\theta$ defines a Markov process over the set of states that is both irreducible and aperiodic and therefore has a unique steady-state distribution. A stochastically stable state is one which has positive probability under the limit of the steady state distribution of the preceding Markov process as $\theta$ goes to zero for any selection$\sigma \in S(G)$. If a state is both a Nash equilibrium of $G$ and a stochastically stable, then it is said to be a stochastically stable equilibrium of $G$.

In what follows, it is assumed that non-elite individuals will coordinate on the stochastically stable Nash equilibrium.

As there is a continuum of non-elite individuals while the definition of stochastic stability presupposes a game with a finite number of players, we proceed as follows. Consider a sequence of finite grids contained in the mass of the non-elite individuals whose limit is the mass of non-elite individuals. Denote such a sequence of finite grids by $\widehat{{N}_{j}}, j\ge 1$. Let ${N}_{j}=\#\widehat{{N}_{j}}$. We call a sequence of finite grids admissible if (i) there is a threshold $\widetilde{{N}_{j}},$ for each $j\ge 1$ such that ${lim}_{j\to \infty }\frac{\widetilde{{N}_{j}}}{{N}_{j}}=\widetilde{\pi }(c)$, (ii) the payoff to a party member is $f\left(\pi \right)\Delta -c$ if the number party members is greater than or equal to $\widetilde{{N}_{j}}$ and is $-c$ otherwise, (iii) the payoff to a non-party member is $\gamma f\left(\pi \right)\Delta$. We say that an equilibrium to be stochastically stable in the coordination game played by the non-elite, it must be the limit of the sequence of stochastically stable equilibria of all admissible sequences of finite grids converging to the mass of the non-elite.

The following proposition characterises which equilibrium is selected when there are multiple equilibria in the non-elite coordination game:

#### Proposition 2.

*Suppose* $0\le \gamma {f}_{max}\Delta <c<{f}_{max}\Delta$ *, then there are two equilibria, one where no non-elite individual joins the party and only the elite have the decision-making power over debt restructuring, and one where all non-elite individuals joins the party and the non-elite have the decision-making power over debt restructuring with probability* ${f}_{max}$ *. The equilibrium where all non-elite individuals join the party is selected when* $\widetilde{\pi }\left(c\right)<\frac{1}{2}$ *.*

#### Proof.

Fix $j$ and consider $\widehat{{N}_{j}}$. For $\theta$ small enough, if at least $\widetilde{{N}_{j}}$ non-elite individuals join the party, then the best response of each non-party member of the non-elite must be to choose to join the party as well. Similarly, if at most $\widetilde{{N}_{j}}-1$ join the party, then the best response of each non-party member must be not to join the party. In states where exactly $\widetilde{{N}_{j}}-1$ join the party, choosing either of the two options, join the party or not join the party, are possible best responses for an individual belonging to the non-elite. It follows that that best responses differ only in states where the number of individuals choosing to join the party is exactly $\widetilde{{N}_{j}}-1$. Now, consider the associated Markov process for small $\theta$. There are two recurrent communication classes (for the definition of the terms "recurrent communication classes", "resistance" and "minimum stochastic potential", see Young (1998)), one where all non-elite individuals choose to join the party (labelled **a**) and one in which all non-elite individuals choose not to join the party (labelled **b**). By Theorem 4 in Young (1998), only states in a recurrent communication class with least resistance will have positive probability weight in the limit of the steady state distribution of the Markov process as $\theta$ goes to zero. Consider the state **b**. Then, (i) there is a best response selection such that given ${N}_{j}-\widetilde{{N}_{j}}+2$ errors, the best response of each individual is to be in **a** and (ii) there is a best response selection such that given ${N}_{j}-\widetilde{{N}_{j}}+1$ errors, the best response of each individual is to be in **a**. Therefore, the minimum resistance of leaving the state **b**, depending on the selection made, is either ${N}_{j}-\widetilde{{N}_{j}}+1$ or ${N}_{j}-\widetilde{{N}_{j}}+2$. It follows that the minimum resistance of a tree oriented from the state **b** to the state **a**, depending on the best response selection made, is either ${N}_{j}-\widetilde{{N}_{j}}+1$ or ${N}_{j}-\widetilde{{N}_{j}}+2$. Next, consider the state **a**. Then, there is both a best response selection such that given $\widetilde{{N}_{j}}-1$ errors, the best response of each individual is to be in **b**, and a best response selection such that given $\widetilde{{N}_{j}}-2$ errors, the best response of each individual is to be in **b**. Therefore, the minimum resistance of leaving the state **a**, depending on the best response selection is either $\widetilde{{N}_{j}}-1$ or $\widetilde{{N}_{j}}-2$. It follows that the minimum resistance of a tree oriented from the state **a** to the state **b**, depending on the best response selection made, is also either $\widetilde{{N}_{j}}-1$ or $\widetilde{{N}_{j}}-2$. The state **b** is the unique stochastically stable equilibrium if and only if both ${N}_{j}-\widetilde{{N}_{j}}+1<{\widetilde{N}}_{j}-1$ and ${N}_{j}-\widetilde{{N}_{j}}+2<\widetilde{{N}_{j}}-2$ or equivalently, both $\widetilde{{N}_{j}}>\frac{{N}_{j}+2}{2}$ and $\widetilde{{N}_{j}}>\frac{{N}_{j}+4}{2}$. As $\frac{{N}_{j}+2}{2}$ $>\frac{{N}_{j}+4}{2}$ if $\widetilde{{N}_{j}}-2>\frac{{N}_{j}}{2}$, the state **a** is the unique stochastically stable equilibrium. Rewriting these inequalities, it follows that state **a** is the unique stochastically stable equilibrium if and only if $\frac{\widetilde{{N}_{j}}-2}{{N}_{j}}>\frac{1}{2}$. For any admissible sequence of finite grids, ${lim}_{j\to \infty } \frac{\widetilde{{N}_{j}}-2}{{N}_{j}}=\widetilde{\pi }(c)$ so that when $\widetilde{\pi }\left(c\right)>\frac{1}{2}$, the unique stochastically stable equilibrium is one where all non-elite individuals do not join the party or conversely, when $\widetilde{\pi }\left(c\right)<\frac{1}{2}$, the unique stochastically stable equilibrium is one where all non-elite individuals join the party. $\square$

Proposition 2 sets out the conditions under which the non-elite, by organising themselves along party lines, engage in collective action to obtain decision-making power over the decision to restructure debt.

First, the probability of successfully usurping decision-making power, conditional on being organised along party lines, is above a certain threshold (${\mathrm{f}}_{\mathrm{max}}>\frac{\mathrm{c}}{\Delta }$). If, to the contrary, ${\mathrm{f}}_{\mathrm{max}}<\frac{c}{\Delta }$ is very low, then even when the non-elite are fully organised along party lines and able to engage in collective action cannot win the decision-making power to restructure debt. Anticipating such an outcome, no non-elite individual will decide to engage in collective in first place and the elite will retain decision-making power.

Second, the cost paid each individual in the non-elite to engage in collective action is below a certain threshold (i.e. $c<{\widetilde{\pi }}^{-1}(\frac{1}{2})$). Note that $c$, the cost to each non-elite individual of engaging in collective political activity, is a measure of how democratic a country is. In a dictatorship, $c$ will be high while in a democracy $c$ will be lower in value. For moderate levels of $c$, in Proposition 2, it is shown that each non-elite individual’s expectations on other non-elite individuals’ most likely course of action is that they will choose to participate; such a belief, when there are multiple equilibrium outcomes, acts as an equilibrium coordination device, inducing the non-elite individuals to their collective action problem. When the $c$ is extremely low in value it becomes a dominant action for each non-elite individual to participate in collective action.

## Policy implications

The impact of a debt crisis on the informal creditors of an insolvent sovereign debtor has been the cornerstone of the contemporary policy debates on how to resolve sovereign debt crisis. These policy measures have been characterised by the twin interventions of austerity through welfare cuts to restore debt sustainability and conditionality attached to official sector bailouts have been a consistent feature of the ad hoc system of debt workouts.

Our analysis differentiates the interests of the domestic elites from those of the domestic non-elite. These elite interests resist debt restructuring required to manage the problem of sovereign insolvency and maintain public investment to protect future domestic income in the aftermath of a negative shock. The involvement of informal creditors as such through a referendum on the decision to restructure debt is efficiency enhancing for all the constituencies affected by a sovereign insolvency.

Our analysis questions the assumption that a sovereign debtor state can be modelled as an agent acting with the trust and confidence of all its citizens. There has been consistent empirical evidence that in the current ad hoc framework, the economic interests of informal creditors and debtor governments diverge (e.g. Stuckler and Basu (2013)).

Our results offer an explanation: by avoiding default and debt restructuring, domestic elites obtain non-contractible benefits that cannot be relied on to repay debt.

The domestic elite has its own economic interests in a debt crisis which are distinct from those of its informal creditors. The debtor state may resist a decision to restructure its debt burden with a view to preserving this benefit. This could entail a significant reliance on official sector bailouts to maintain debt sustainability.

In the Eurozone crises, the contrasting response of Iceland and Greece to an economy-wide negative shock offers the different roles that informal creditors can play in crises resolution. In the case of Greece, the 1st referendum was called off in response to market pressures and the second referendum was called to approve a bailout rather than a debt restructuring. This raises issues about the design of institutions to enhance the role of informal creditors a point explicitly raised in a recent debt workout guide made by UNCTAD that explicitly accounts for the role of informal creditors at several points in the lead up to a debt restructuring.

The motivation behind the UNCTAD roadmap was the “socialisation of losses from private debts and the subsequent emergence of sovereign debt crisis in developing and developed countries.” (UNCTAD, 2015) The proposal aims to enhance “coherence, fairness and efficiency of sovereign debt workouts.” (UNCTAD, 2015). The efficiency deficit identified in the proposal arises from the problem of restructurings which are ‘too little too late’. The proposals set out ‘specific recommendations for each step of a sovereign debt workout.

A key aspect of each recommendation is the explicit recognition and acknowledgement of civil society (“informal creditors”) as an independent constituency whose interests are both distinct from those of the debtor government and the formal creditors. For instance, the principle of impartiality recognises that debt workouts need to be defined by a “neutral perspective particularly with regard to sustainability assessments and decisions about restructuring terms” (UNCTAD, 2015) rather than as a procedure to fulfil the self-interest of debtors and creditors. Further the issue of “sustainability requires that sovereign debt workouts are completed in a timely and efficient manner…while minimising costs for economic and social rights and development in the debtor state.” (UNCTAD, 2015) Here for debt workouts to restore debt sustainability, ex-ante and interim efficiency must be achieved. This limits the problem of “too little too late” by explicitly accounting for the impact of workouts on informal creditors.

**Fig. 1:**
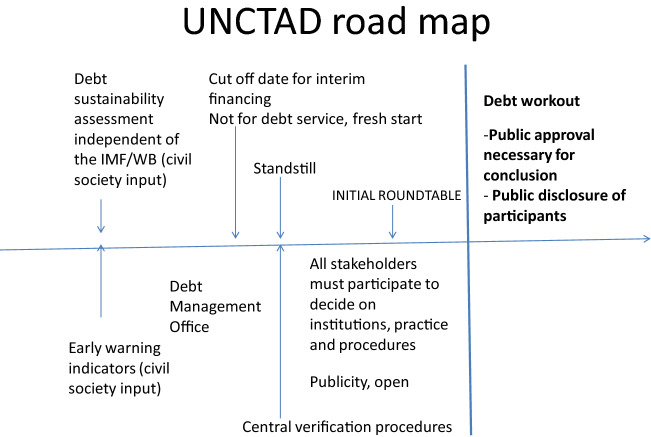
UNCTAD road map and guide

Following this account, the road map articulates a clear role for civil society intervention through repeated referenda at key points in the lead up to a debt workout. The referenda are the proposed as a mechanism independent of the formal contractual arrangements between a debtor and its creditors. There are two ways in which the road map is informed the non-contractibility of elite payoffs. First there is a recognition that there is a common interest between the debtor state, controlled by an elite with decision-making power over restructuring debt) and its formal creditors that leads them into consensual contractual arrangements. Second, this interest is not shared by the non-elite (the informal creditors)—hence the need for referenda and independent informal creditor intervention to achieve ex ante and interim efficiencies. The several points at which these efficiency claims can be assessed are set out in Fig. 1 below.

The UNCTAD roadmap specifies formal opportunities for inclusion the non-elite in the decision to restructure debt. Importantly, for the UNCTAD roadmap to be effective the process by which informal creditors would be consulted in the decision to restructure debt needs to be laid out ex ante.

Our results suggest that for the UNCTAD roadmap to work in practice, the institutions it creates must increase ${f}_{max}$ and lower $c$. Only then will the domestic non-elite collectively organise to grab the opportunity to obtain decision-making power over debt restructuring. By anticipating such an outcome, the interest charged by formal creditors ex ante will be lower as well.

## Conclusion

This paper specifies the conditions in which the involvement of informal creditors through a referendum enhance the efficiency gains of debt workouts. The paper formalises the problem of “too little too late”. It makes a case for giving informal creditors a voice in the lead up to and in debt renegotiations. Giving informal creditors a voice at the point at which debt becomes unsustainable and the at point at which debt restructuring negotiations are under way will make a difference to two problems- the problem of delay in recognising a problem of debt sustainability and the problem of debt workouts being too shallow requiring repeated official sector involvement in the restoration of debt sustainability through bailouts.

In the context of the wider debate, this paper raises but does not answer the following questions. To what extent should considerations of equity and fairness limit the official sector involvement to restore liquidity and prevent a self-fulfilling insolvency crisis? Does the informal creditor participation at this stage limit the ability of the official sector to intervene to stop a self-fulfilling crisis?

One answer to this question depends on the nature of the crisis that is triggering the intervention. If the intervention is an isolated self-fulfilling liquidity crisis, then there is justifiably a limited role of private sector bail-ins. However, if the cost of this official sector intervention is conditionality that imposes an excessive cost on the informal creditors, then in addition to the nature of the crisis, would require a solution that involves a debt workout- in which the costs of a crisis are distributed equitably between informal and formal creditors. This would require an examination of the claims of informal creditors in a sovereign debt workout—which goes beyond giving the informal creditors a voice in debt restructuring negotiations, a topic for future research.
